# Rethinking multiscale cardiac electrophysiology with machine learning and predictive modelling

**DOI:** 10.1016/j.compbiomed.2018.10.015

**Published:** 2019-01

**Authors:** Chris D. Cantwell, Yumnah Mohamied, Konstantinos N. Tzortzis, Stef Garasto, Charles Houston, Rasheda A. Chowdhury, Fu Siong Ng, Anil A. Bharath, Nicholas S. Peters

**Affiliations:** a ElectroCardioMaths Group, Imperial College Centre for Cardiac Engineering, Imperial College London, London, UK; b Department of Aeronautics, Imperial College London, South Kensington Campus, London, UK; c National Heart and Lung Institute, Imperial College London, South Kensington Campus, London, UK; d Department of Bioengineering, Imperial College London, South Kensington Campus, London, UK

**Keywords:** Cardiac electrophysiology, Cardiac arrhythmia, Electrogram, Machine learning, Predictive modelling, Deep learning

## Abstract

We review some of the latest approaches to analysing cardiac electrophysiology data using machine learning and predictive modelling. Cardiac arrhythmias, particularly atrial fibrillation, are a major global healthcare challenge. Treatment is often through catheter ablation, which involves the targeted localised destruction of regions of the myocardium responsible for initiating or perpetuating the arrhythmia. Ablation targets are either anatomically defined, or identified based on their functional properties as determined through the analysis of contact intracardiac electrograms acquired with increasing spatial density by modern electroanatomic mapping systems. While numerous quantitative approaches have been investigated over the past decades for identifying these critical curative sites, few have provided a reliable and reproducible advance in success rates. Machine learning techniques, including recent deep-learning approaches, offer a potential route to gaining new insight from this wealth of highly complex spatio-temporal information that existing methods struggle to analyse. Coupled with predictive modelling, these techniques offer exciting opportunities to advance the field and produce more accurate diagnoses and robust personalised treatment. We outline some of these methods and illustrate their use in making predictions from the contact electrogram and augmenting predictive modelling tools, both by more rapidly predicting future states of the system and by inferring the parameters of these models from experimental observations.

## 1 Introduction

Cardiac arrhythmias, particularly atrial fibrillation (AF), are a major global healthcare challenge in the developed world. Arrhythmias describe the abnormal and self-perpetuating propagation of action potentials (AP) within the myocardium. Their initiation and maintenance are incompletely understood and this has hindered the development of effective and reliable therapy. Treatment for AF is often through catheter ablation, where the regions of myocardium determined to be responsible for initiating or perpetuating the disturbance are targeted and made electrically inactive through the localised application of radio-frequency energy or freezing. For paroxysmal AF, catheter ablation delivers relatively good outcomes, with success rates in the region of 80–90 percent [1]. However, outcomes of catheter ablation in patients with persistent AF remain disappointing, and is effective in only approximately 50 percent of patients, despite all forms of adjunctive ablation strategies [2].

Identifying the critical sites responsible for abnormal AF maintenance has been a major focus of research, with a number of driving mechanisms, including rotors [3], multiple wavelets [4] and epi-endo disassociation [5], being proposed. Recent clinical studies, such as the CONFIRM study [6], have tested the new approaches of catheter ablation by targeting the foci of rotational drivers, with initially promising results showing that 86% of 101 cases achieved AF termination or slowing. However, subsequent studies suggest more moderate outcomes with Steinberg et al. [7] reporting only 4.7% of 47 cases achieved AF termination, while 60% documented recurrence within 12 months. The efficacy of this technique may be in part due to the poor spatial resolution of the global mapping catheter used [8]. Techniques involving the targeting of complex fractionated atrial electrograms (CFAE) [9], high dominant frequency (DF) [10] and singularities identified during phase mapping [11] have each been used as strategies for terminating arrhythmias. However, none of these adjunctive ablation strategies have been shown to add any value to the conventional approach of electrically isolating the pulmonary veins [2]. Part of the reason for this may be that they each discard a large proportion of the information content of the acquired electrogram signals or their spatio-temporal association during analysis. Additionally, not all identified sites may be critical to the perpetuation of the arrhythmia, leading to excessive ablation. The complexity of the underlying electro-architecture of myocardium therefore requires a more sophisticated, personalised and multi-faceted approach to address the challenge of treating AF.

The principle data modality used clinically for the treatment of AF is the contact electrogram, which arises from the superposition of electric fields induced by charged ions moving across cell membranes in the myocardium. It is the electrical signature of action potential propagation through tissue which implicitly encodes the functional and structural characteristics of the local substrate. The electrogram therefore provides a wealth of information which is rarely fully utilised in current clinical practice. Electrograms are normally only broadly categorised by binary descriptors – such as *simple* or *complex*, *early* or *late* [12,13], fractionated or non-fractionated – with much of the signal content effectively discarded. Despite a number of studies based on interpreting clinical electrogram data [14,15], these do not investigate how electrogram morphology is influenced by individual electro-architectural factors. Our knowledge about the direct effects of electrical remodelling on electrogram morphology is consequently poor, considering the number of these abnormalities related to cardiac diseases [16]. Leveraging the electrogram to infer electroarchitectural properties of the myocardium may therefore provide new direct insight in locating critical sites for ablation.

Multiple concurrently recorded electrograms may be combined to evaluate the spatio-temporal propagation patterns occurring in the tissue. This activity can also be inferred from the surface of the body [17]. More recently, predictive modelling of action potential propagation is emerging as a potential tool for personalised testing and optimisation of interventions [18], but this technology is heavily dependent on the accuracy of the underlying calibration of parameters. This can only be achieved by fully leveraging the huge wealth of information now available clinically. The data science revolution in the form of sophisticated machine learning algorithms and increasing availability of computing power, opens up possibilities to manage this data overload, both in terms of learning from the data, inferring model parameters and consequently making increasingly accurate predictions.

### 1.1 Machine learning in cardiac electrophysiology

Machine learning describes a class of algorithms which *learn* model parameters from a set of training data (for which outcomes may, or may not, be known) with the purpose of accurately predicting outcomes for previously unseen data. Training data that includes associated outcome labels can be used for *supervised* learning in which the algorithm uses this knowledge to directly improve its prediction. In contrast, *unsupervised* learning seeks patterns in the data with more limited guidance, of which clustering is a common example. Although there is considerable overlap, machine learning methods are considered to differ from more conventional statistical modelling, such as regression, in that they are more concerned with the predictive accuracy of the resulting model rather than the ability to explain the reasoning behind its parameters and determining concrete relationships between the data. The high accuracy of some of the more recent machine learning methods – which are virtually impossible to analyse analytically – has justified this lack of transparency.

All machine learning algorithms seek some form of mapping that models the relationship between input data and outcome. In an abstract context, we suppose that we have a model *f*, governed by one or more parameters θ, which maps an input x to some output y, under the relation

(1) $$f\left(x,\theta \right)=y\text{.}$$

The form and dimensions of x and y in Equation (1) are a function of the particular problem under consideration. For example, x may be a large one-dimensional vector (time-series) in the case of a music-classification problem, or a two-dimensional image in the case of object recognition. For regressions, the output y may be a prediction of the dependent quantity, while for classification problems, y is usually a label which assigns the corresponding input to a single class. The size of θ depends on both the problem and also on the chosen model. For example, for a linear regression between two variables θ would consist of only two values (namely the *slope* and *intercept*), while for a many-layered deep neural network with high-dimensional input data, the size of θ may be of $O\left(10^{6}\right)$ or more.

Broadly speaking, the process of *training* a supervised machine learning algorithm is the notion of seeking θ such that, for some set of training input data {x} with corresponding outcomes {y}, a given *loss function* is minimised. While there are a number of loss functions, each with their own properties [19], a simple loss function might be the $L_{1}$ loss function which computes the differences between the predicted outputs and the actual outputs and is given by

(2) $$\varepsilon =\overset{N}{\underset{i=1}{\sum }}||f\left(x_{i},\theta \right)-y_{i}|{|}_{1}.$$

If sufficient (and suitable) training data are used with an appropriate model, the expectation is that the model will then correctly predict the outcomes for other inputs which did not form part of these data.

Supervised machine learning is increasingly being used in medicine [20]. One area of cardiac electrophysiology in which machine learning has become particularly prevalent to date is the analysis of the Electrocardiogram (ECG), in part due to its wide availability and its potential to conveniently provide important information about cardiac function without intervention. There now exists a substantial body of literature on the application of machine learning tools to classify ECGs. A review of some of the earlier work is given by Ref. [21]. The recent PhysioNet challenge to classify single-lead ECG segments into four categories (sinus rhythm, AF, other rhythm or too noisy) has catalysed developments in this area [22]. Most approaches require some form of preprocessing of the signal, including de-noising and correcting for baseline wander. While convolutional neural networks [[23], [24], [25], [26], [27], [28], [29], [30]] and recurrent neural networks [23,31] are gaining popularity, many studies still achieved accurate classification results using other algorithms such as ensembles of decision trees (random forests) [32,33], multi-level binary classifiers [34] and least-squares support vector machine classifiers [35]. The use of these approaches in combination also provides accurate classification [24,31]. Recently, online real-time feature extraction and classification of ECGs using machine learning is being explored [36] and similar approaches are being used to diagnose more specific cardiac abnormalities [37].

In contrast, relatively little attention has been given to applying machine learning to make predictions from the contact intracardiac electrogram, or to predict the spatio-temporal patterns of activation in myocardium. Recently, there have been studies to characterise AF using *in silico* or clinical contact electrograms [14,15], as well as for the automated location of *in silico* re-entrant drivers using electrograms [38].

### 1.2 Predictive numerical modelling

Numerical modelling assumes the system under observation obeys particular physical laws, known *a priori* and often represented in the form of partial differential equations, which are used to predict the future state of the system given an initial state. These equations often contain a number of parameters, which are estimated from experimental observations or experience.

While predictive modelling has advanced significantly in the field of cardiac electrophysiology for the past decade, only recently have the numerical methods and clinical imaging technologies improved sufficiently to allow viable predictions to be made on anatomically accurate geometries [39,40]. However, challenges still remain in how to accurately personalise and validate these models, as well as how to safely incorporate them into clinical practice.

### 1.3 Outline

In this review, we describe some of the opportunities machine learning can provide in the field of cardiac electrophysiology. We illustrate these through examples as well as discuss their potential impact on arrhythmia management. We begin with the contact electrogram the data modality on which much of modern clinical electrophysiology is based. We introduce machine learning approaches to analysing and classifying these signals, contrasting both feature-based methods and deep neural networks, and show how they can be used to potentially elucidate a wealth of electro-architectural information about the myocardial substrate. We then discuss recent advances by our group in modelling action potential propagation and how machine learning might supplement and extend these methods to improve our ability to create personalised models which can be used on clinically relevant timescales.

## 2 Feature-based classifiers

*Features* describe characteristics of a process being observed. They are often represented in a numerical form and together form a feature vector. A feature-based machine learning algorithm then uses these feature vectors as input during both training and prediction. The selection of informative features is critical to the effectiveness of a machine learning algorithm to predict the correct output label. When a large number of features are available algorithms may struggle to generalise due to redundancy of information between features. Feature selection algorithms can alleviate this issue by selecting a subset of features which promote learning and improve the ability of the algorithm to make accurate predictions. The identification of which features are important in specific situations may also generate hypotheses to motivate further investigation of mechanistic links.

When the output label is one of a finite set of possible discrete values, the algorithm is termed a classifier. In the simplest case of binary classification, the accuracy of a learning algorithm may be characterised by a number of statistical measures. *Sensitivity* describes the percentage of positive outcomes that are predicted as positive, while *specificity* captures the proportion of negative outcomes predicted as negative. *Positive (and negative) predictive value* instead captures the proportion of positive (and negative) predictions, which are truly positive (and negative).

A broad range of feature-based classifiers for supervised machine learning exist, and we refer the reader to previously published comprehensive reviews for specific details [41,42]. Both linear and non-linear classifiers map the input features to a set of classes *d* using a weighted sum,

$$d\left(x\right)=f\left(\underset{i}{\sum }w_{i}{\left(\phi \left(x\right)\right)}_{i}\right)$$

with the weights, $w_{i}$ learnt during training. In the linear case ϕ(x)=x. The function *f* maps the result of the sum onto the different classes and may be a simple threshold function in the case of a binary classification, or probability densities more generally. While in general not as accurate as non-linear classifiers, linear classifiers are typically faster and so may be more effective in time-critical applications [43].

Several approaches may be used to determine the weights of linear classifiers. Linear discriminant analysis [44] seeks weights which best separate inputs x of different classes. Support vector machines (SVMs) [45] instead seek to maximise the margin between a hyperplane and the two data classes it separates. The *k*-nearest neighbour classifier is a non-linear classifier which uses the classes of the nearest training samples to predict the classification for unseen samples [46]. Decision trees [42] approach the classification problem feature-by-feature with branches in the tree representing different values a feature can assume. The leaves of the tree denote the final classification.

The performance of the above predictors can often be improved using the method of bootstrap aggregation, or *bagging* [47]. Rather than training a single predictor on a training dataset, a number of training datasets are generated by drawing observations at random but with replacement and predictors are trained on each of these *bootstrap* datasets. When making a prediction, the results of these predictors are *aggregated* – usually by voting when performing classification – to form the final predictor. Random forests [48] extend the bootstrap aggregation of decision trees by additionally selecting random subsets of features when deciding how to split at each node. These approaches help to overcome the problem of over-fitting often present with decision trees where they fail to generalise.

### 2.1 Application to electrogram classification

We explore the use of supervised machine learning to classify individual electrograms based on the presence of cellular abnormalities. For initial proof of concept we use signals acquired from cell monolayers in culture. While distinctive from clinical electrograms, they enable us to assess the capabilities of these algorithms in a controlled context and ensure signals can be labelled accurately. We investigated the hypothesis that controlled functional modulations of the monolayers can be accurately and precisely predicted from the recorded unipolar electrogram morphology using supervised machine learning methods. In particular, we sought the classification of electrical signals according to pharmacological gap junction uncoupling.

#### 2.1.1 Data acquisition and pre-processing

Electrogram recordings were acquired as previously described [49]. In brief, cell monolayers of neonatal rat ventricular myocytes (NRVMs) were seeded onto five microelectrode arrays (MEA), each consisting of 60 electrodes (MultiChannel Systems, Reutlingen, Germany). Ten-second recordings were made at a sampling frequency of 25 kHz while pacing from one edge, before and after administration of 40 μM carbenoxolone (CBX) to increase gap junction uncoupling. No signal filtering was applied during data acquisition. All animal procedures were conducted according to the standards set by the EU Directive 2010/63/EU.

**Fig. 1:**
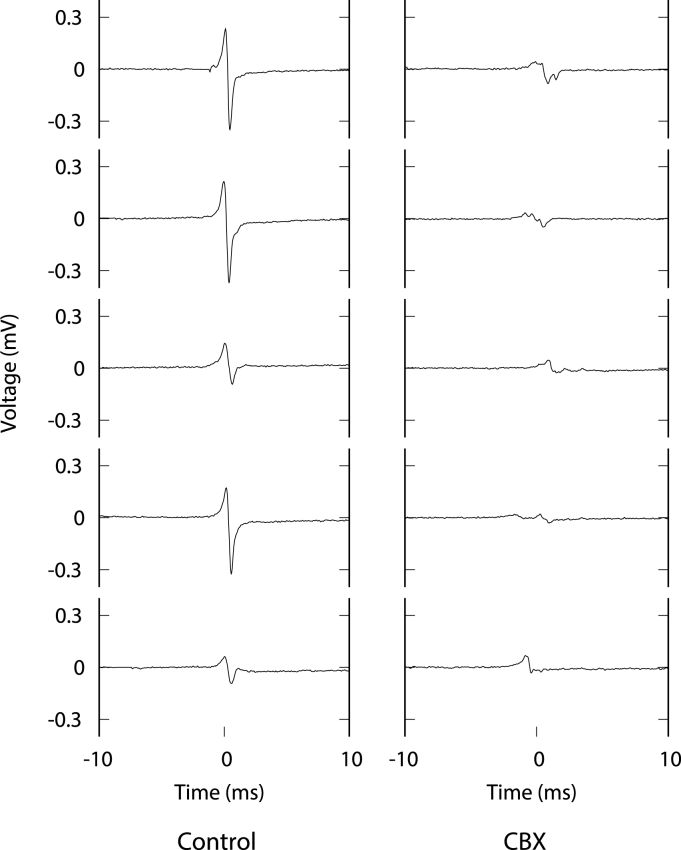
Examples of 20 ms segments of electrogram recordings from the Control and CBX groups, after removal of the stimulus artefact.

Pacing artefacts were removed by approximating the exponentially decaying stimulus deflection with a rational polynomial and subtracting it from the recorded signals. The dataset was further curated to remove recordings from electrodes where no further deflections were present. This resulted in 485 control electrograms and 471 electrograms after treatment with CBX. The dataset was partitioned into a training dataset and a testing dataset. The testing dataset consisted of all electrograms recorded from one plate (90 control and 104 CBX electrograms). The training dataset consisted of the remaining four plates (395 control and 367 CBX electrograms). Examples of electrograms from the control and CBX classes are shown in Fig. 1.

#### 2.1.2 Feature extraction

The high sampling rate of the electrograms would result in very high-dimensional input data if used directly. We mapped each signal onto a pre-defined feature space of much lower dimension. Each electrogram recording was represented by a fixed set of 27 time-, frequency- and morphological-based features, extracted from the signal using a custom-written algorithm (Matlab R2017b). Details of these features are provided in the Supplementary Material. Sequential Forward Selection (SFS) [50] was used to select a subset of these features which were sufficient to differentiate the control and CBX classes. In brief, SFS is a *bottom-up* approach to choosing discriminatory features. Starting with an empty *feature set*, the algorithm sequentially adds features from the candidate set of features which maximises a given objective function, until the addition of further features provides no improvement. Classification accuracy is used as the objective function. Feature selection indicated that only three of the 27 features considered were sufficient to distinguish the control and CBX classes: *electrogram amplitude*, *standard deviation of the autocorrelation function* and the *scale with minimum energy* in the continuous wavelet transform of the signal. The set of values from the selected electrogram characteristics form the feature vector for that electrogram. These feature vectors were subsequently used to train the classifier.

The bootstrap aggregating (or *bagging*) ensemble tree method [47] was applied during both feature selection and classification training.

#### 2.1.3 Results

**Fig. 2:**
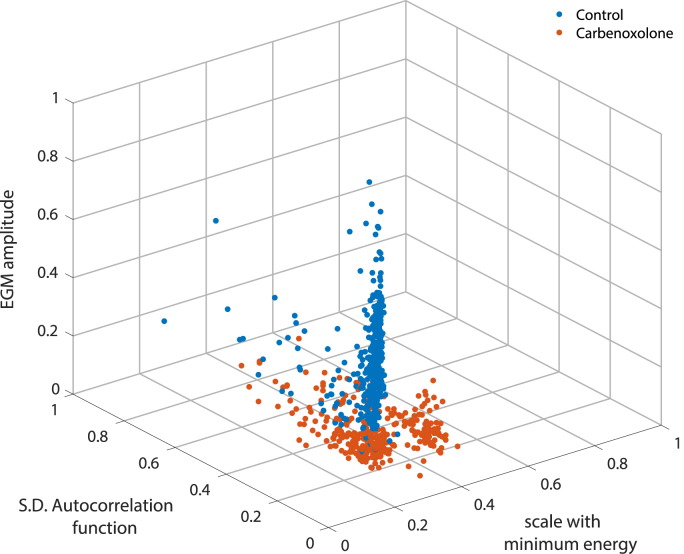
3D scatter plot of the most relevant features, normalised in the interval [0,1], as determined by SFS. No single feature clearly discriminates between the control and carbenoxolone classes.

Fig. 2 shows the distribution of all electrogram feature vectors from the training dataset in the corresponding three-dimensional feature space. It is evident that no single feature alone effectively discriminates between the two classes.

**Fig. 3:**
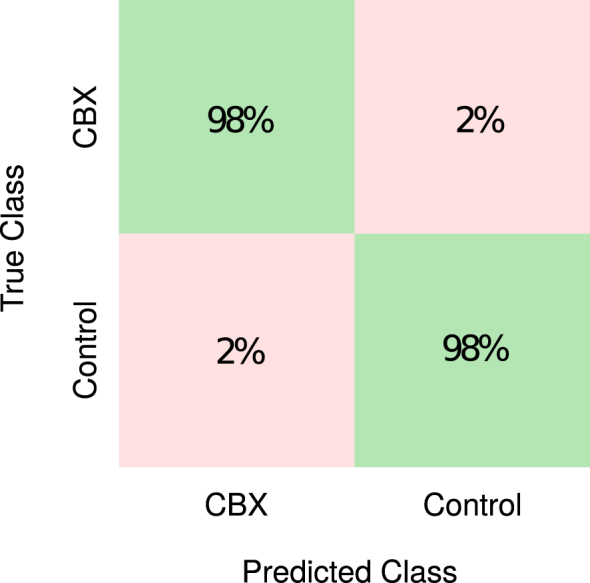
Confusion matrix comparing the predictability of classes using the training dataset. Diagonal cells (green) show the percentage of electrograms that were correctly classified.

Validation was performed on the training data using ten-fold cross-validation. A total of 30 decision trees, with a leaf size of one, were used for the bagging ensemble method. The performance characteristics of the trained model are given in Table 1. A specificity of 98.1% was achieved on the training data when using only the three features chosen by SFS. This is illustrated by the *confusion matrix* shown in Fig. 3, which compares predicted class against true class. This indicates the model was capable of accurately distinguishing the classes. The model performance was then measured using the unseen test dataset of 194 electrograms. It achieved a 96.7% sensitivity, 84.4% specificity, 83.8% positive predictive value and 96.8% negative predictive value, indicating the model has generalised successfully.

**Table 1:** Performance of classification training using the Bagging Ensemble method and evaluation of the subsequent prediction model on the test dataset.

	Classification training (734 EGMs)	Prediction model testing (194 EGMs)
Sensitivity	98.1%	96.7%
Specificity	98.3%	84.4%
Positive predictive value	98.4%	83.8%
Negative predictive value	97.7%	96.8%
Error rate	1.9%	10%

The computational time of the training process was also measured. Calculation of the electrogram features was the dominant cost, at 18.84 ± 5.2 seconds per electrogram. The time taken for the bagging ensemble method to train a model using the feature vectors was 3.25 ± 0.13 seconds.

## 3 Convolutional neural networks

One of the limitations of conventional machine-learning techniques is their inability to be applied directly to high-dimensional data, necessitating a transformation into a suitable feature-space representation that captures characteristics of the data relevant for discrimination, while remaining invariant to irrelevant aspects. As illustrated in Section 2.1.2, extracting these features often requires significant domain-specific knowledge and expertise in order to hand-engineer suitable algorithms, and thus produce an informative representation that supports discrimination. This process can, however, be circumvented if the feature-extraction step is automated.

Representation learning [51] encompasses a set of techniques within the field of machine learning which enable a model to automatically learn and discover for itself discriminating features directly from the raw observational data. Among the most popular of current techniques are layered artificial neural networks, which take inspiration from neuroscience. They are composed of artificial neurons (simplified versions of biological neurons) arranged in layers, where the neurons in one layer are connected to many, if not all, of the neurons in the subsequent layer. The artificial neuron is a non-linear mapping from an input value to an output value. The output values from all the neurons in one layer are each multiplied by adjustable parameters, called weights, to form a weighted sum as input to one individual neuron in the subsequent layer. A neural network is, therefore, a complex system of weighted non-linear functions nested within each other and it is these weights that must be learnt in order for the network to accurately map raw input data to a desired output.

Neural networks learn their own weights during training: initially the weight values are randomly selected and thus when a model is given input it is initially highly unlikely to predict the correct output label. During training, the model is shown raw input data and the associated label or output value. For each input example the model makes a prediction based on the current weight values, and the error between the prediction and the true desired output is measured. The weights are then modified in order to minimise this error via a process called back-propagation, the details of which are provided in Ref. [52]. An *epoch* is defined as a complete pass over the training data. Unlike the discriminant classifier of Section 2.1 which only needs one pass, neural networks benefit from multiple passes over the training data. With sufficient training data and sufficient iterations (epochs) over all the data the weights converge onto values that enable the model to make accurate predictions for the training dataset. The trained model is subsequently tested on a validation dataset it has never before seen in order to measure its predictive power.

The weights of a neural network can be seen as the features of the learnt representation, automatically discovered without the need for manually-designed feature detectors. When the network contains several layers in between the input layer and the final output layer – known as *hidden* layers – it is referred to as a deep network, from which the term deep-learning arises. These models are therefore representation-learning methods with multiple non-linear layers, each transforming the representation, beginning with the raw input, into increasingly more discriminative representations.

Today, deep-learning techniques provide state-of-the-art solutions in the fields of object recognition [53,54] and detection [55], speech recognition [56] and natural language processing [57,58], and are increasingly being used in other domains such as genomics [59] and challenging segmentation problems required for geometric reconstruction in biomedical imaging [60]. Recently, studies have applied deep neural networks to the ECG signal [[23], [24], [25], [26], [27], [28], [29], [30]]. These studies have all made use of convolutional neural networks (or *convnets*).

Fully-connected neural networks treat neighbouring data points identically to those spaced far apart. In the case of time-series data, they do not account for the temporal structure and autocorrelation that may be present in the raw input data. As such, they may fail to recognise, for example, a QRS complex in an ECG if it had been shifted in time by half a beat compared to the training data examples. Convnets are deep-learning architectures that cater to this need for translation-invariance. They exploit compositional hierarchies – whereby higher-level features are generated by accumulating a set of lower-level features – often exhibited in datasets derived from the natural world.

### 3.1 Application to classifying electrograms

**Fig. 4:**
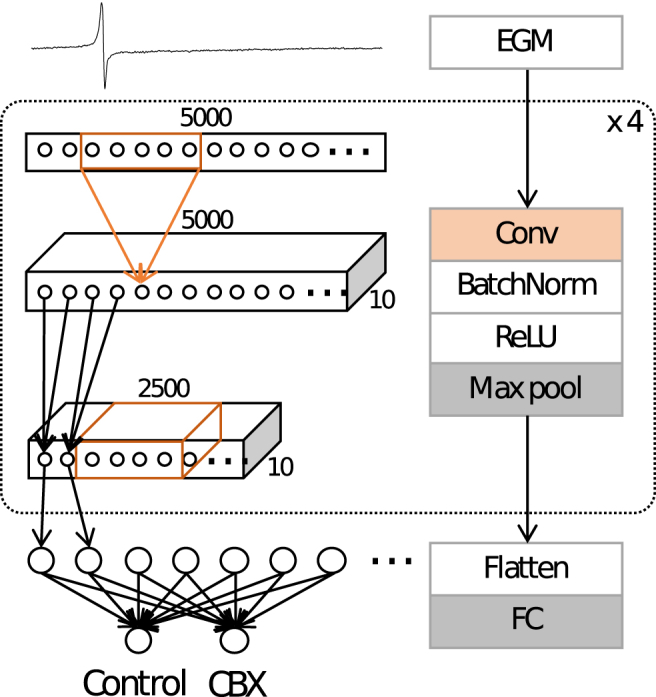
Schematic of the convolutional neural network.

We demonstrate the application of convolutional neural networks, using the same data as described in Section 2.1.1, to perform the classification directly from the labelled time-series data. The network is composed of four repeated blocks, each itself consisting of a convolutional layer, a batch normalisation layer [61], a non-linear ReLU layer [62] and a max pooling layer [63]. Batch normalisation adjusts the inputs to a layer to have mean zero and standard deviation of one, and is a technique for improving the stability of the network. The ReLU layer is a form of activation function, while max pooling layers are used to down-sample the input as it progresses through the network. The last block is followed by one fully-connected layer to the two output classes. A schematic of the architecture is shown in Fig. 4.

#### 3.1.1 Training

During training, a randomly selected one-second segment (thereby guaranteed to contain one deflection) was taken from the full ten-second recording and down-sampled to 5 kHz. Training was carried out for 250 epochs using the Adam optimiser [64] with a variable learning rate starting at 0.0001 and a weight-decay of zero. The error was evaluated using the cross-entropy loss function [19], and training was repeated five times each with different random initial weights to evaluate the performance (averages and standard deviations), giving a measure of the robustness of the architecture and optimisation process. This was carried out using the PyTorch framework [65] on an GTX 1080ti GPU (NVIDIA Corporation, USA).

Cross-validation was carried out to measure the robustness of the prediction model as well as to aid tuning of the parameters of the learning algorithms and design choices of the architecture (e.g. number of layers). Once these hyper-parameters were sufficiently tuned, the validation and training data were combined (four plates) and the model retrained. It was then evaluated using the test data (one plate). Trained models were evaluated by splitting the ten-second recordings from the test or validation datasets into ten segments corresponding to the ten deflections and classifying each segment. If six or more deflections were classified correctly, the recording was considered successfully classified and the uncertainty of this positive classification was computed using the binary entropy function. In practice, there was little variation between the ten deflections within a signal, resulting in consistent classification of each recording.

#### 3.1.2 Results

**Fig. 5:**
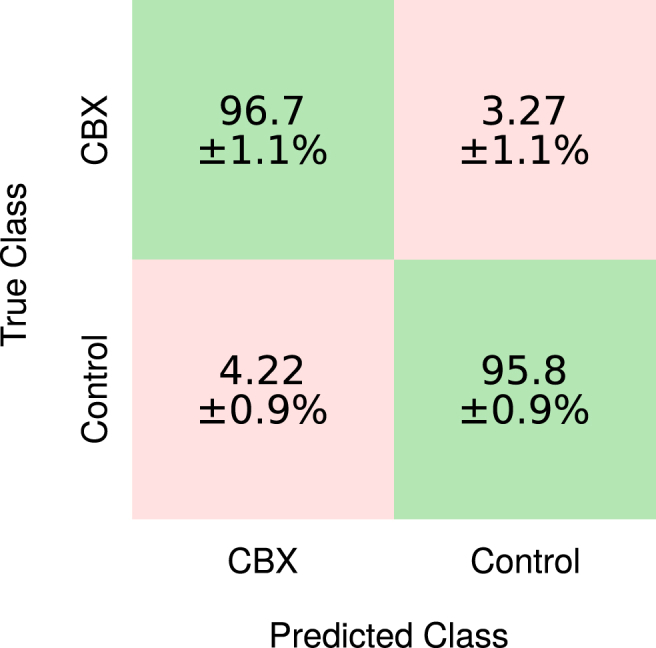
Confusion matrix from the binary classification of electrograms before and after administration of carbenoxolone to monolayers of cultured myocytes.

Table 2 shows the classification accuracy using four-fold cross-validation after all design choices were made. The average accuracy across the folds was 96.2% with a standard deviation of 1.5%, indicating a reliably robust model, and in general the entropy values remained low at approximately 0.05, indicating that each ten-second segment was consistently classified correctly or incorrectly. The standard deviation of the five repeats for each fold showed the models were converging to a similar optimum state regardless of the initial weight values. The final evaluation of the model on the test data resulted in an overall accuracy of 96.3 ± 0.7%, with results of 96.7 ± 1.1% sensitivity, 95.8 ± 0.9% specificity, 96.4 ± 0.7% positive prediction value and 96.2 ± 1.2% negative predictive value. The confusion matrix for this binary classification problem is shown in Fig. 5.

**Table 2:** Total classification accuracy results from the 4-fold cross-validation and final testing. For each model, the convolutional neural network was trained a total of five times to ensure the model was robust to differences in random initialisations. Averages and standard deviations of the classification accuracy are presented.

	Fold	Classification accuracy (%)
Cross-validation	1	96.7 ± 0.8
	2	97.2 ± 0.6
	3	96.7 ± 1.0
	4	94.0 ± 0.7
**Testing**		**96.3**±**0.7**

## 4 Numerical modelling

Numerical modelling predicts the future behaviour of a dynamical system from a known state under an *a priori* belief in the physical laws governing the system. In particular, it can allow observations to be extrapolated forward in time to help better understand the likely behaviour of a physical system and, by modulating the parameters and initial condition of the system appropriately, allow hypothetical scenarios to be explored *in silico*. This makes it a potentially invaluable tool for both improving our understanding of the mechanisms driving arrhythmogenesis and as a direct clinical tool for aiding diagnosis and planning intervention.

### 4.1 Tissue-scale continuum modelling

The physical processes responsible for the cardiac action potential in a cardiac myocyte are a complex choreography of ion movements across the cell membrane. Mathematically, these are often described by systems of ordinary differential equations (ODEs) which range in complexity from two equations to more than twenty [66]. The parameters of these equations have been chosen based on fitting the individual equations to experimental measurements. Cells are electrically coupled through gap junctions, which can be mathematically modelled as resistors. However, modelling the whole heart at a cellular scale is computationally intractable. Consequently, homogenisation of the discrete cell model leads to a bi-domain continuum model in the form of two partial differential equations (PDEs),

(3) $$\nabla \cdot \left(\sigma_{i}\nabla v\right)+\nabla \cdot \left(\sigma_{i}\nabla v_{e}\right)=\chi \left(C_{m}\frac{\partial v}{\partial t}+I_{ion}\right),$$

(4) $$\nabla \cdot \left(\sigma_{i}\nabla v\right)+\nabla \cdot \left(\left(\sigma_{i}+\sigma_{e}\right)\nabla v_{e}\right)=0,$$

supplemented with appropriate boundary conditions [67]. Here *v* is the transmembrane potential, $v_{e}$ is the extracellular potential, $C_{m}$ is the membrane capacitance per unit area, *χ* is the cellular surface-to-volume ratio and $I_{ion}$ is the transmembrane current density from the coupled action potential ODE model. Anisotropy and heterogeneity of the myocardium is captured in the intracellular conductivity tensor $\sigma_{i}$ and extracellular conductivity tensor $\sigma_{e}$. With the further assumption of equal anisotropy ratios in these spaces, such that $\sigma_{i}=\lambda \sigma_{e}$, this system of equations can be further reduced to a single PDE, again with appropriate boundary conditions, known as the monodomain model,

(5) $$\frac{\lambda }{1+\lambda }\nabla \cdot \left(\sigma_{i}\nabla v\right)=\chi \left(C_{m}\frac{\partial v}{\partial t}+I_{ion}\right)$$

A comprehensive review of the mathematical models used in the cardiac electrophysiology domain is given by Clayton et al. [67]. The monodomain model belongs to the class of reaction-diffusion PDEs. The diffusion component can be considered to relate to the biophysical process of ion propagation through gap junctions between cells, while the reaction component is the cumulative result of the action potential model describing the opening and closing of ion channels in the membrane (gating variables) and related ion movements into, or out of, the cell.

### 4.2 Numerical methods for action potential propagation

To solve equation (5) in all but the most trivial of scenarios requires the use of numerical approximations. The continuous PDE is transformed into a system of algebraic equations which are more amenable to solution on a computer. This transformation may use one of several discretisation techniques, such as finite difference, finite element or spectral approximations and with sufficient spatial and temporal resolution can provide very accurate approximations to the true solution of the PDE. However, the wide range of time-scales on which the different physical processes in the model occur makes the numerical solution of this system challenging and may lead to long simulation times even with considerable computational resources.

One technique being explored within our group for modelling electrophysiology is the spectral/hp element method [68]. This approach combines the flexibility of the finite element method to model, for example, the complex geometry of the heart chambers, with the numerical benefit of spectral methods, by enriching the polynomial space of each element with higher-order basis functions. In particular, this allows an approximation of comparable accuracy to a conventional finite element discretisation to be achieved with a smaller algebraic system of equations, resulting in faster simulations and ultimately a shorter time to solution. We have also explored the simulation of action potential propagation in the left atrium using a surface representation of the chamber wall [69], further reducing the size of the numerical problem to solve.

Even with these advancements, the computational cost of using numerical methods to accurately perform predictive modelling is still substantial, with the time-to-solution being orders of magnitude higher than what might be required for an interactive clinical tool. Furthermore, the inference of model parameters is highly challenging and even more computationally costly. In Section 5 and Section 6 we consider opportunities for machine learning techniques to complement modelling and help address these difficulties.

## 5 Recurrent neural networks

Deep neural networks can be trained to predict the future behaviour of a dynamical system [[70], [71], [72]], and subsequently their internal representation can be used to estimate the latent parameters of the system. Neural networks have been shown to be faster (at inference time) than commonly used numerical simulation approaches [70,71]. Indeed, while they require large amounts of data to train, once the optimal network weights have been found, obtaining predictions from unseen inputs requires only a fraction of the time and computational resources in comparison to conventional numerical methods. Despite the drawback of generating only approximate solutions, leveraging the fast prediction performance of neural networks may enable large numbers of *what-if* scenarios to be rapidly explored in a fraction of the time of conventional numerical modelling. Clinically, this might allow the viability of potential therapeutic strategies to be quickly tested and accelerate the calibration of more precise numerical modelling which can be used to further optimise the treatment.

Recurrent neural networks differ from purely feed-forward neural networks, such as the convolution neural networks considered in Section 3, in that they are designed for processing sequences of inputs. They incorporate a feedback loop where the output of each step in the sequence is added to the input of the next step [63]. These types of networks are therefore well suited to dealing with data sequences. This allows information to be propagated along the sequence: each output will then be conditioned not only by the current input in the sequence, but also by all previous inputs. However, in practice, vanilla recurrent neural networks can only store information for a short number of steps. Long short-term memory (LSTMs) networks are a particular variation of recurrent neural networks developed to alleviate this problem and allow the learning of longer term dependencies [63].

### 5.1 Application to predicting two-dimensional diffusion

Here, we present a proof of principle study where we apply this approach to a two-dimensional diffusion problem with a spatially heterogeneous and anisotropic diffusion tensor. Diffusion is a key component of models of excitable media, such as cardiac tissues [67]. Our system is governed by the following equations:

(6) $$\frac{\partial v\left(x,y,t\right)}{\partial t}=\nabla \left(D\left(x,y\right)\nabla v\left(x,y,t\right)\right),\left(x,y,t\right)\in \text{Ω}\times \left[0,T\right],$$

(7) $$v\left(x,y,0\right)=v_{0}\left(x,y\right),\left(x,y\right)\in \text{Ω},$$

(8) $$v\left(x,y,t\right)=0,\left(x,y,t\right)\in \partial \text{Ω}\times \left[0,T\right].$$

Here, D is a diagonal 2 × 2 matrix with non-zero diagonal elements $d_{0}$ and $d_{1}$, governing diffusion on the horizontal and vertical axis, respectively. The computational domain $\text{Ω}={\left[-2,2\right]}^{2}$.

#### 5.1.1 Training data generation

Numerical simulations were performed using a regular mesh of 80×80 square elements, using a modified Legendre polynomial basis with polynomials up to order P=5. The solution over time was subsequently sampled on a regular 64×64 grid for input into the neural network. A total of 1600 numerical simulations were performed using Nektar++ [68] with initial condition and diffusion fields drawn at random from a predefined distribution. The initial condition consisted of spatially smoothed noise. This was created by first generating a random spatial frequency spectrum in the Fourier domain. This had a two-dimensional frequency profile drop-off as ${\left(f_{x}^{2}+f_{y}^{2}+f_{c}\right)}^{\alpha /2}$, with alpha randomly chosen to be either −1 or −2 and $f_{c}=3$. The quantities $f_{x}$ and $f_{y}$ are measured in terms of cycles per domain length. Furthermore, there is a sharp cut-off at $f_{x}^{2}+f_{y}^{2}<f_{o}^{2}$, with $f_{0}=8,12$ or 16 which is randomly drawn independently for each simulation. The spatial frequency profile was multiplied by standard Gaussian noise and phases were drawn from a uniform distribution between 0 and 2*π*. Finally, a symmetric version of the spectrum is inverted to obtain the initial condition in the spatial domain and then normalised to achieve a certain level of contrast. All initial conditions are gradually smoothed to zero when approaching the boundary of the domain. Moreover, they were first generated at a resolution of 128 × 128 pixels and then interpolated onto the mesh used for the simulation.

The diffusion field was characterised by six parameters. A line with random orientation (*θ*) and location (*β*) is chosen to partition the domain, with one part denoted as healthy and the other as scarred tissue. The former is given a higher diffusion coefficient with respect to the latter. Diffusivity in the domain is therefore characterised by four different diffusion parameters: $d_{0}$, $d_{1}$, $d_{0,scar}$, $d_{1,scar}$, which are chosen to satisfy the conditions,

(9) $$\frac{\text{max}\left(d_{0},d_{1}\right)}{\text{min}\left(d_{0},d_{1}\right)}=\frac{\text{max}\left(d_{0,scar},d_{1,scar}\right)}{\text{min}\left(d_{0,scar},d_{1,scar}\right)}\equiv \gamma ,$$

(10) $$\frac{d_{0}}{d_{0,scar}}=\frac{d_{1}}{d_{1,scar}}\equiv \lambda .$$

Here, the anisotropy ratio *γ* and heterogeneity ratio *λ* are randomly drawn from a uniform distribution on the intervals [1,3] and [2,7], respectively. Finally, for each simulation we randomly selected which of $d_{0}$ and $d_{1}$ was assigned to have the highest magnitude. This consequently determines the direction along which diffusion is fastest. The direction of fastest diffusion was assigned a value drawn from the interval [3.2, 3.8].

#### 5.1.2 Network architecture

**Fig. 6:**
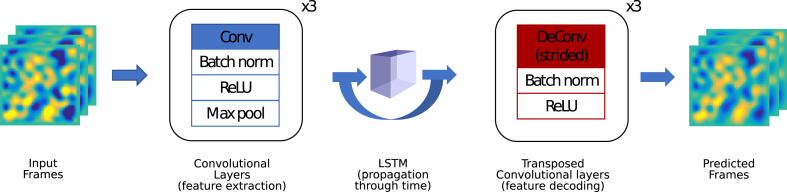
The architecture of the network used to predict the future behaviour of the 2D diffusion system.

To predict the future behaviour of the system, we built a fully convolutional neural network consisting of three main blocks, similar to the architecture used by Ehrhardt et al. [72]. First, a three-layer encoding network extracts relevant features from the input frames, while performing dimensionality reduction. By compressing the information that passes through the layers, this encoder network thus acts as a bottleneck that encourages the network to only extract a useful representation of the system. While Ehrhardt et al. [72] used a portion of a pre-trained VGG network [73], here we train the network end-to-end to tailor the features extracted to the specific physical system under consideration. Next, a convolutional LSTM layer [74] progresses the features in time for as many steps as necessary. After the recurrent layer, the structure of our predictive network is completed by a three-layer decoder network that transforms the output of the LSTM back into frames in the spatial domain. Transposed convolutions are used with a stride of two [75] to return to the original resolution. Batch normalisation [61] and ReLU non-linearities [63] were used after each convolutional layer in both the encoder and the decoder. A schematic of the neural network can be found in Fig. 6, while Table 3 has a summary of the key network parameters. The hyper-parameters of the network were chosen manually, after a brief exploration of the hyper-parameter space. Therefore, it is possible that a more thorough search would further improve prediction accuracy.

**Table 3:** Details of the architecture for the prediction network. The number of channels in the last layer of the decoder is variable because it depends on how many frames are requested as output (K).

	Nb. of channels	Filter size	down-/up-sampling
Encoder	(64,32,32)	(5,5,5)	Max-pooling (2 × 2)
LSTM	32	5	n.a.
Decoder	(32,64,K)	(4,4,4)	Stride = 2

#### 5.1.3 Network training

The network received as input 2 or 3 sequential frames and was trained to predict the next 11 frames, while only a smaller number of target frames (variable between 1 and 7) was used to back-propagate the prediction error, and thereby constituting useful information for the network training process. All networks considered in this study were able to extrapolate for more time steps than that used during training. We used Mean Squared Error (MSE) – the average of the squared difference between targets and predictions – as the loss function, and trained the network for 1000 epochs using Adam update schemes [76], weight decay [61] and a batch size of 64. The learning rate started at 0.0005 and was decreased by a factor of ten after 700 epochs. The simulations were split into training and testing using a 5-fold cross-validation scheme. In addition, 20% of the training dataset was set aside for validation, since the final model was chosen as that which exhibited best performance across epochs on this validation dataset. The training was carried out using the PyTorch framework [65] and required between 40 minutes and 60 minutes per network on a GTX 960 GPU (NVIDIA Corporation, USA), depending on the number of back-propagated frames.

#### 5.1.4 Results

Our aim was to investigate the changes in prediction accuracy with varying amounts of training data, quantified as the number of frames given as the input ($K_{b}$) and as the target ($K_{t}$) to the network. The former determines how much information the network can use to make predictions while the latter, which corresponds to the number of back-propagated target frames, is used to calculate the error which informs the update of the network weights. The more frames that are used for back-propagation, the more future time steps can be aggregated to build the error signal that guides the network learning process. The disadvantage is that longer simulations are required to train the network and there is an increased risk of over-fitting.

**Fig. 7:**
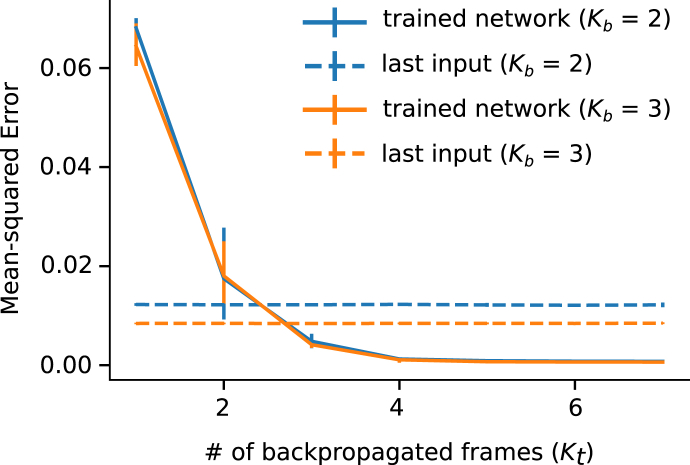
Accuracy of next steps prediction versus number of input ($K_{b}$) and back-propagated ($K_{t}$) frames. Mean Squared Error (MSE), averaged over the test dataset, first, and the 5 cross-validation folds, then, against $K_{t}$ and for $K_{b}=2,3$. Error bars extend to the minimum and maximum MSE among the 5 folds. Dashed lines represent the last input level.

**Fig. 8:**
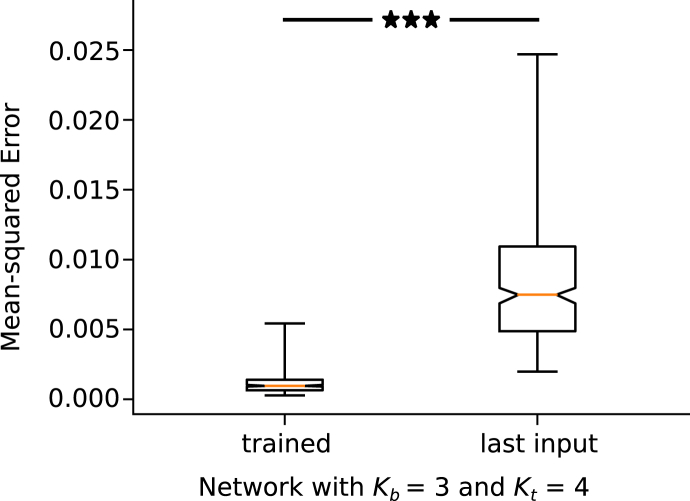
Comparison between the full MSE distribution across the test dataset for the predictions of a single trained network, compared with that given by using the last input frame as the prediction. The asterisks represent statistical significance with p-value $<10^{-4}$ (Wilcoxon signed rank test).

Prediction errors on the test dataset are shown in Fig. 7, for networks trained with different numbers of input and target frames. The solid lines correspond to the MSE averaged over the test dataset and over the five-fold cross-validation, with the error bars extending from the minimum to the maximum accuracy achieved across the five folds. The MSE is plotted against the number of back-propagated target frames, but is computed as an average across the prediction of 11 future frames, for all networks shown. The dashed lines represent the “last input” level, computed as the error that would be achieved if the last input frames were used as the predictions. This control test provides a baseline against which to judge the generalisation capability of the network. Fig. 8 shows the full “last input” level distribution against the performance achieved by one of the trained networks ($K_{b}=3$, $K_{t}=5$). It can be observed that the network is making effective use of the information it receives as input, since the accuracy achieved is better than that obtained with no physical knowledge for each individual simulation in the test dataset.

To assess statistical significance, we compared the two distributions using a Wilcoxon signed rank test: we obtained a p-value smaller than $10^{-4}$ against the null hypothesis of equality of medians, as shown in Fig. 8. Such a null hypothesis can be interpreted as the situation in which the neural network makes advantageous use of its inputs, but has not learned the physics behind it. Furthermore, the plot in Fig. 7 suggests that the learning capabilities of the network saturate after a certain number of back-propagated target frames (potentially from $K_{t}=4$). From that point onwards, the network is able to maintain similar error levels when extrapolating the predictions further ahead in time. This could be indicative of the network having better assimilated the mechanics of diffusion. The benefit of using a smaller number of back-propagated frames would be evident when there is a limited time available to run the simulations necessary to build the training dataset: in this case, being able to train effectively with shorter, rather than longer, simulations is advantageous.

**Fig. 9:**
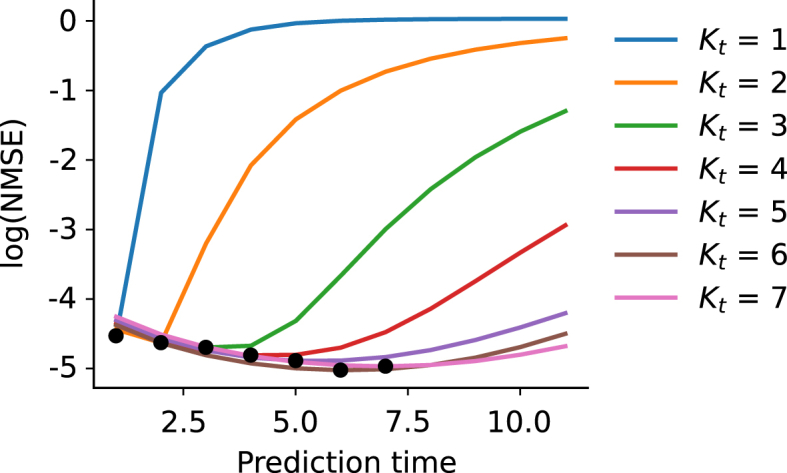
The logarithm of the Normalised Mean Squared Error (NMSE) against the prediction time for networks trained with various $K_{t}$ (and $K_{b}=3$). The black dots represent the $K_{t}$ corresponding to each line.

It is possible, however, that the gain in prediction accuracy shown in Fig. 7 is influenced by the progressive decrease over time of the overall energy of the system, caused by the diffusion dynamics. To control for this, we computed the normalised mean squared error (NMSE) – the MSE between predicted and target frames divided by the $L_{2}$ -norm of the target frames at each individual time point. Such a measure is similar to the fraction of variance explained by a regression algorithm, and allows us to directly compare performance at different time steps. Fig. 9 shows the logarithm of NMSE as a function of time for networks trained with $K_{b}=3$ and different values of $K_{t}$, averaged over the test dataset and the cross-validation folds. It can be noted that, at each time step, the NMSE is inversely proportional to the number of back-propagated frames, suggesting that networks with a higher $K_{t}$ are able to explain a larger proportion of the variability of all future frames.

Once trained, each prediction from the neural network took less than one second to calculate, compared to 40 seconds required by numerical modelling. Extending this to models of cardiac electrophysiology, such a gain in computational speed would allow for a quicker exploration of multiple scenarios of interest, such as the alteration of functional and structural characteristics of different areas of the myocardium when planning intervention.

### 5.2 Application to estimating diffusion parameters

Robustly and accurately estimating the parameters for models is critical for them to be useful in prediction. In this section we consider an example of how machine learning can meet this need. Extending the next-step prediction of Section 5.1, we explored how accurately the parameters of the diffusion model can be estimated from one of the networks trained for predictions. We specifically considered the network with $K_{b}=3$ and $K_{t}=4$, as shown in Fig. 8.

We first extracted the internal representation of the network for all 11 predicted future frames. Specifically, this is the activity of the LSTM units. This multidimensional vector was used as the input to a second neural network with two convolutional, and one fully connected, layers which was trained to predict the six parameters $d_{0}$, $d_{1}$, $d_{0,scar}$ and $d_{1,scar}$, as well as the orientation (*θ*) and location (*β*) of the boundary between healthy and scarred tissue. Details of this network are given in Table 4.

**Table 4:** Details of the architecture for the parameters estimation network.

	Channels/units	Filter size	down-/up-sampling
Convolutional	(128,64)	(6,6)	Stride = 2
Fully connected	(6)	n.a.	n.a.

#### 5.2.1 Results

**Fig. 10:**
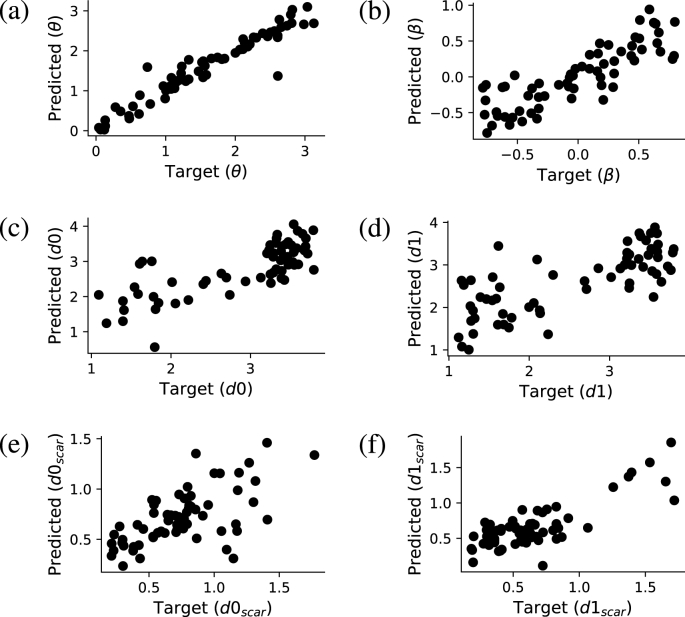
Accuracy of parameter inference from the internal representation of the prediction network with $K_{t}=4$ and $K_{b}=3$ for (a) boundary angle *θ*, (b) boundary position *β* and (c–d) scar parameters $d_{0}$, $d_{1}$, $d_{0,scar}$ and $d_{1,scar}$. The predicted parameter values are plotted against the target values for a subset of the test dataset.

Results are shown in Fig. 10. The correlation coefficients between target and predicted parameter values are 0.84, 0.80, 0.80, 0.70, 0.95, and 0.87 for $d_{0}$, $d_{1}$, $d_{0,scar}$, $d_{1,scar}$, *θ* and *β*, respectively. Despite there being potential for improvements, the achieved accuracy indicates that the internal representation learnt by the network does contain information about physically relevant quantities. This suggests that our deep learning model is able to assimilate at least some of the mechanisms intrinsic to the physical system under consideration.

## 6 Approximate Bayesian Computation (ABC)

Approximate Bayesian Computation (ABC) is a statistical inference technique that can be applied as a parameter fitting method to incorporate uncertainty estimates into the fitting process [[77], [78], [79]]. The algorithm interrogates the range of outputs of a numerical model by drawing different parameter choices from a defined *prior* probability space, running the simulation, and comparing the output to experimental data. This probability space is then sequentially refined based on a distance function which measures the closeness of the simulated output to the experimental output. After multiple iterations (or reaching a chosen error threshold), the algorithm produces a discrete approximation to the true *posterior* probability distribution of model parameters given the observed experimental data. The distributions give more information to a modeller on the ability of the experimental data to constrain the parameter choice and the resulting credibility of the full model.

### 6.1 Application to inferring cell model parameters

Tissue-scale cardiac electrophysiology simulations are built on models of the action potential of single myocytes. These *cell models* are solved by calculating the opening and closing kinetics of transmembrane and internal ion currents, and their effect on the membrane potential of the cell. Each parameter in ion current sub-models is chosen specific to the particular cell type. The parameter values are based on data from patch-clamp experiments which interrogate the dynamics of specific ion currents in isolated myocytes at a range of prescribed voltages [80].

The standard approach is to fit ion channel parameters to these data using a traditional method such as least squares regression. These methods produce point estimates and thus do not take into account uncertainties introduced through the fitting process itself when multiple parameter choices can result in similar values of the fitting loss criterion. This has led to discrepancies between cell models which purport to represent the same cell type [81].

Daly et al. previously investigated the use of ABC on parameters chosen in the original Hodgkin-Huxley action potential model [78,82]. They found that parameters were generally well constrained by the experimental data. These data were average current traces which inform both voltaic and temporal behaviour of a channel. Modern experimental studies predominantly report steady-state behaviour of channels in response to voltage steps.

#### 6.1.1 Neonatal rat ventricular sodium channel

We investigate the ability of modern patch-clamp data, which may contain less information, to constrain parameters of a physiological model for neonatal rat ventricular myocytes (NRVMs) [83]. We present the result of fitting the fast sodium channel of the NRVM model using ABC. The fast sodium channel plays a crucial role in the generation of a cellular action potential and the propagation of an electrical signal through cardiac tissue; consequently, it is critical to have confidence in any *in silico* model of the channel. We use the ABC Sequential Monte Carlo (ABCSMC) algorithm with a population adaptation strategy from the *pyabc* python library (http://pyabc.readthedocs.io/en/latest/) [79] and the *myokit* python library (http://myokit.org) for running simulations [84]. The equations for the fast sodium channel [83] include three *gates*: activation, fast inactivation, and slow inactivation, and are given by,

(11) $$I_{Na}=G_{Na}m^{3}hj\left(V-E_{Na}\right)$$

(12) $$m_{\infty }={\{1+\text{exp}\left[\left(p_{1}+V\right)/p_{2}\right]\}}^{-1}$$

(13) $$j_{\infty }=h_{\infty }={\{1+\text{exp}\left[\left(q_{1}+V\right)/q_{2}\right]\}}^{-1}$$

(14) $$\tau_{m}={\left\{\frac{p_{3}\left(V+p_{4}\right)}{1-\text{exp}\left[p_{5}(V+p_{4})\right]}+p_{6}\text{exp}\left(-V/p_{7}\right)\right\}}^{-1}$$

where the parameters $p_{1}$ - $p_{7}$ and $q_{1},q_{2}$ are determined from experimental data, the original published values of which are given in the second column of Table 5. The gates govern the proportion of open channels in the cells and thus affect the maximum current that is able to flow across the membrane. These equations were adapted for NRVMs from earlier cell models of different species by varying only the channel conductance [83]. We therefore use only the directly applicable data for observations in the ABCSMC algorithm. These data are from patch clamp experiments on adult rat ventricular cells [85,86]. They include five patch clamp protocols testing activation, inactivation and recovery characteristics of the channel. The protocols do not explicitly test temporal characteristics of the current, and thus we retain the temporal parameters of the fast and slow inactivation processes to reduce the dimensionality of the problem. For each of the nine parameters that we constrain using ABCSMC, initial priors were set to uniform distributions roughly an order of magnitude larger than the parameter setting in the original model.

**Table 5:** Results of ABCSMC inference for the parameters of the fast sodium channel.

	orig.	prior	posterior		
			mean	min	max
p1	45	(0, 100)	43.4	43.2	43.6
p2	−6.5	(-50, 0)	−11.6	−11.6	−11.5
p3	0.235	(0, 1)	0.0717	0.0707	0.0726
p4	47.1	(0, 100)	79.7	79.3	79.9
p5	−0.1	(-50, 0)	−36.2	−50.0	−12.2
p6	0.0588	(0, 1)	0.00345	0.00302	0.00373
p7	11.0	(0, 1000)	682	325	998
q1	76.1	(0, 100)	72.5	72.9	73.1
q2	6.07	(0, 50)	9.54	8.82	10.0

#### 6.1.2 Results

Table 5 also shows the prior distribution ranges and statistics of the posterior distributions. Seven of the nine parameters appear well constrained by the data, of which four ($p_{1}$, $p_{2}$, $q_{1}$, $q_{2}$) are relatively close to the original values. These parameters govern the steady-state activation and inactivation (both fast and slow) of the current, confirming that the patch clamp protocols predominantly test the steady-state characteristics of this current.

**Fig. 11:**
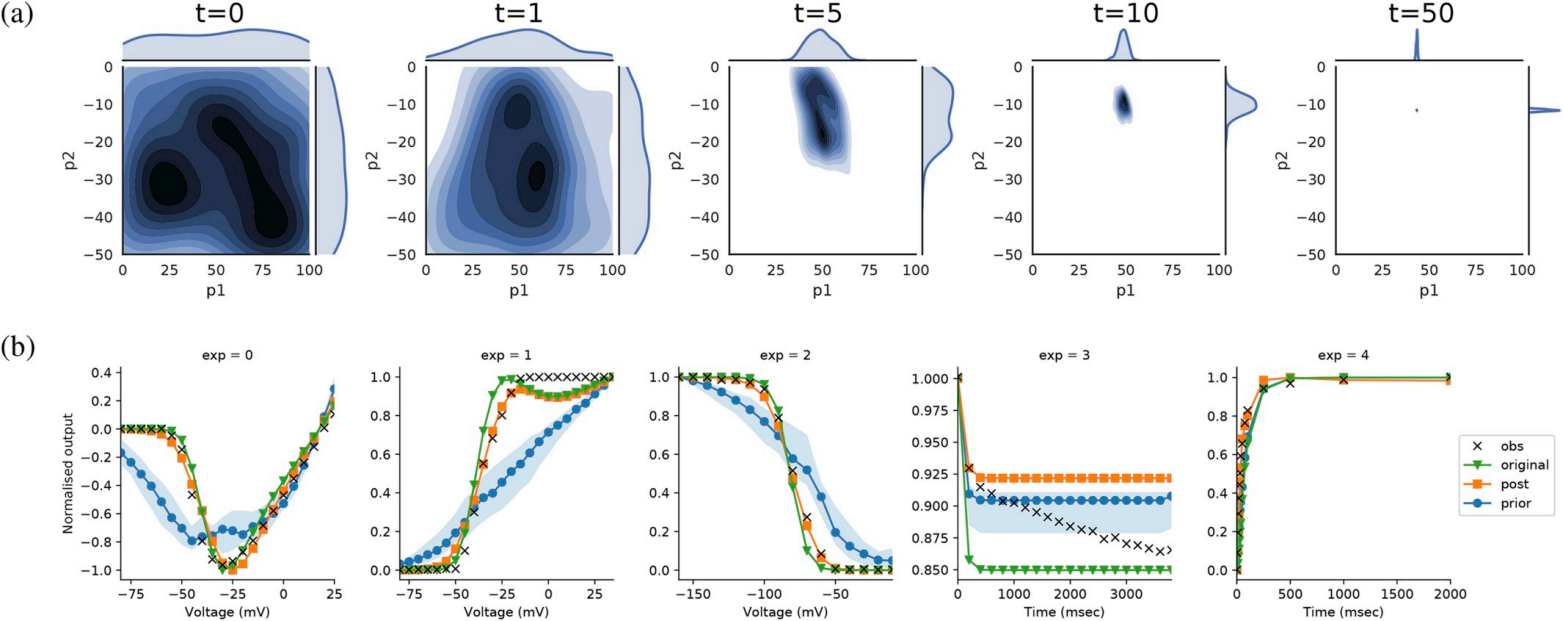
(a) Kernel density estimates for steady-state activation parameters showing sequential constraining of distributions. (b) Data used to fit channel (black crosses) are plotted for each patch clamp experiment. Simulation results are also plotted for original parameter settings (green triangles), 100 samples from prior distribution (blue circles), and 100 samples from posterior distribution (orange squares). Shaded area indicates 95% confidence intervals around the median line.

Fig. 11(a) shows how the distributions of the two steady-state parameters of activation are sequentially constrained through the iterations of ABCSMC. Fig. 11(b) shows the data used to fit the current, along with simulation results using original parameters, 100 prior distribution samples and 100 posterior distribution samples. The output for the posterior distribution is close to both the original settings and the experimental data. In some aspects, particularly the upper half of inactivation behaviour in exp = 2, the ABCSMC fit is a noticeable improvement over the original parameter choices. However, in other areas such as the start of activation (seen in exp = 0 and exp = 1), the ABCSMC fit is further from the observed data than the original settings. For exp = 3 which tests the normalised peak current of a regular train of voltage pulses, the equations of the model appear unable to capture both the initial exponential and then linear decay of the observed data, shown by the fact that both the ABC posterior and original parameter choices end in a constant relationship after the initial decay portion.

Despite the large variation present in two of the parameters, the posterior results in Fig. 11(b) show little variation. This indicates that the current patch-clamp protocols may not sufficiently interrogate temporal aspects of the channel, as both unconstrained parameters (p5 and p7) govern this aspect of the model equations. This highlights the value of the ABC approach; using traditional fitting methods, we would not be aware of the unidentifiability in these parameters. More complex patch-clamp protocols could be investigated in an attempt to improve the ability of the data to constrain the model.

## 7 Discussion

In this review we have shown the potential benefits of a number of machine learning approaches and how they can enable us to extract more information from the data we collect. The electrogram is the ubiquitous data modality of the cardiac electrophysiology catheter laboratory and yet the relative information content extracted from these complex signals is currently poor. We have demonstrated that by quantifying and combining a range of features in the signal, some of which are already used in isolation, and applying machine learning algorithms to them we can learn more about the properties of the underlying myocardium and the substrate that sustains arrhythmias. Deep learning allows us to further automate this process, removing the inherent bias of manually choosing potentially sub-optimal features, and allowing the neural network to extract latent representations which best discriminate between classes directly from the signal.

Numerical modelling is becoming increasingly established within the cardiac electrophysiology field, due to the increased availability of computational power, and improved resolution of clinical imaging technologies. However, the numerical resolution requirements for action potential propagation and complexity of ion channel kinetics still necessitates high computational cost. Furthermore, the number of parameters and difficulties associated with deriving appropriate values experimentally or clinically means that great care is needed when incorporating these into predictive modelling. We have shown how machine learning can help in both inferring appropriate parameter values from data as well as quantifying how certain we can be in those parameter values and therefore how confident we can be in the model output.

### 7.1 Limitations

In analysing the micro-electrode array electrogram data in Section 2 the large-amplitude stimulus artefact, created by pacing of the culture, was first removed. This was required for the feature-detection algorithms to reliably measure the electrogram characteristics used to form the feature vector. Since the stimulus artefact dominates the signal, it was also a necessary pre-processing step for the convolutional neural network approach. Without its removal the network was unable to distinguish specific morphological features of the response signal.

For the robust training of deep neural networks, used in both Section 3 and Section 5, the volume of data and computational cost of training is high. While data augmentation techniques are used to improve the generalisation of models, additional recorded data would further improve the quality of the predictions made by these methods.

Graphics Processing Units (GPUs) are particularly effective at undertaking the learning process for deep neural networks and their use is essential to produce trained models in tractable timescales. In this context, feature-based classifiers provide a performance advantage in situations where appropriate features are known and can be defined *a priori* to distinguish the classes. However, the computational time for prediction using the trained models is negligible for both feature-based and deep-learning based methods.

## 8 Conclusions

Cardiac arrhythmias are a major global healthcare problem and there is significant scope for improving their diagnosis and treatment. Improvements will be achieved from better understanding of the mechanisms sustaining fibrillation, as well as increasingly personalised treatment. Modern machine learning techniques and numerical modelling, when applied appropriately, both have great potential to help fulfil this role and their combination, in particular, offers a powerful approach to achieving personalisation of care.

## Conflict of interest

None declared.
