# Supplementary material for: Rethinking multiscale cardiac electrophysiology with machine learning and predictive modelling
Source: Comput Biol Med. 2019 Jan;104:339–51. doi: 10.1016/j.compbiomed.2018.10.015 (PMC6334203; doi:10.1016/j.compbiomed.2018.10.015)
Supplement: Multimedia component 1. [file mmc1.pdf]

Supplementary Material

Rethinking multiscale cardiac electrophysiology with machine learning and predictive modelling

Chris D. Cantwell, Yumnah Mohamied, Konstantinos N. Tzortzis, Stef Garasto, Charles Houston, Rasheda A. Chowdhury, Fu Siong Ng, Anil A. Bharath, Nicholas S. Peters

Electrogram feature extraction

A total of 27 features were initially calculated for the feature-based machine learning approach. Each ten-second signal contained ten deviations from baseline as a wavefront propagated across the electrode during 1Hz pacing. In the control preparations, these primarily consist of a positive deflection (R-peak), followed by a negative deflection (S-peak), corresponding to the approaching and receding wavefront, respectively. The R-peak of a complex was defined as the point of maximum potential above a 60% threshold (based on the maximum amplitude of the whole signal). This was chosen to eliminate small deflections due to baseline noise and secondary deflections due to fractionation. The corresponding S-peak was defined as the minimum potential within 100 samples following an R-peak. The electrogram amplitude feature was subsequently defined as the potential difference between corresponding R- and S-peaks, averaged across all complexes in the signal. Similarly the time between the R and S peaks, the length of the whole QRS complex, width of the R and S peaks and time between the QRS onset and S peak were used as features. Gradients of the segments from baseline to the R peak, between the R and S peak and following the S peak back to baseline were also used. Finally, ratios between the R and S amplitudes and widths were included.

A second-order bandpass filter (0.05-10Hz) and QRS removal was used to help identify the T-wave. Subsequently, the field potential duration [1] and time between QRS onset and the mid-point of the T-wave were used as features. T-wave amplitude was also included. The sample autocorrelation function (ACF) [2] measures the correlation, $acf(k)$, between time series y_t and y_{t+k}, for $0 < k < N_S$, with $N_S = 250,000$ being the total number of samples in the signal. The *standard deviation of the ACF* feature was then defined as the standard deviation of $acf(k)$. Other time-domain features included fractionation index and frequency, Shannon entropy [3] and logarithmic energy entropy [4].

The electrogram signal was transformed using the continuous wavelet transform (CWT), based on a Morlet wavelet. The CWT is a time-frequency analysis which convolves the signal with scaled forms of the mother wavelet. The *scale with minimum/maximum energy* were then defined as the scales which contains least/most energy in this wavelet decomposition [5]. Other feature computed from the transformation included the *percent small-scale energy* and the *variance of energy*. Dominant frequency [6] was also computed.

Examples of next-frame prediction

The supplementary videos show examples of predicting the evolution of the diffusion problem described in Section 5.1. In each video, the left panel shows the target frames (sampled from the numerical simulation). The centre panel shows the corresponding frames predicted by the neural network. The right panel shows the mean-squared error between the prediction and target for each frame. Examples are shown for different initial conditions and heterogeneous spatial distributions of diffusion coefficients.

References

[1] P. Camelliti, S. A. Al-Saud, R. T. Smolenski, S. Al-Ayoubi, A. Bussek, E. Wettwer, N. R. Banner, C. T. Bowles, M. H. Yacoub, C. M. Terracciano, Adult human heart slices are a multicellular system suitable for electrophysiological and pharmacological studies, Journal of molecular and cellular cardiology 51 (3) (2011) 390–398.

[2] S. G. Guillén, M. T. Arredondo, G. Martin, J. M. F. Corral, Ventricular fibrillation detection by autocorrelation function peak analysis, Journal of electrocardiology 22 (1990) 253–262.

[3] C. E. Shannon, A mathematical theory of communication, Bell Syst. Tech. J. 27 (1948) 379–423.

[4] S. Aydın, H. M. Saraoğlu, S. Kara, Log energy entropy-based EEG classification with multilayer neural networks in seizure, Annals of biomedical engineering 37 (12) (2009) 2626.

[5] M. Talbi, A. Aouinet, R. Baazaoui, A. Cherif, Ecg analysis based on wavelet transform and modulus maxima, International Journal of Computer Science Issues (IJCSI) 9 (1) (2012) 427.

[6] R. Telgarsky, Dominant frequency extraction, arXiv preprint arXiv:1306.0103 .
